# Reviewer acknowledgement 2015

**DOI:** 10.1186/s40168-016-0188-8

**Published:** 2016-08-09

**Authors:** Jacques Ravel, Eric Wommack

**Affiliations:** 1 Department of Microbiology and Immunology, University of Maryland School of Medicine, Institute for Genome Sciences (IGS), Baltimore, MD US; 2 Delaware Biotechnology Institute, University of Delaware, Newark, DE US

## Abstract

The editors of *Microbiome* would like to thank all our reviewers who have contributed to the journal in 2015.

**Rachel Adams**

United States of America

**Emma Allen-Vercoe**

Canada

**Katherine Amato**

United States of America

**Florent Angly**

Australia

**Claire Arrieta**

Canada

**Manimozhiyan Arumugam**

Denmark

**Guy Baele**

Belgium

**Martin Iain Bahl**

Denmark

**David Baltrus**

United States of America

**Kenneth Barfod**

Denmark

**Christine Bassis**

United States of America

**Kirsten Benkendorff**

Australia

**Gabriele Berg**

Austria

**David Berry**

Austria

**Kyle Bibby**

United States of America

**Kristi Biswas**

New Zealand

**Cinnamon Bloss**

United States of America

**Blaise Boles**

United States of America

**Robert Britton**

United States of America

**Brandon Brooks**

United States of America

**Dries Budding**

Netherlands

**Tsute Chen**

United States of America

**Nicholas Chia**

United States of America

**David Coil**

United States of America

**Maria Carmen Collado**

Spain

**William Cookson**

United Kingdom

**Emily Cope**

United States of America

**Michael Cox**

United Kingdom

**Keith Crandall**

United States of America

**Leah Cuthbertson**

United Kingdom

**Karen Dannemiller**

United States of America

**Robert Dickson**

United States of America

**Sharon Donovan**

United States of America

**Sylvia Duncan**

United Kingdom

**Juliana Durack**

United States of America

**Robert Dyer**

United States of America

**Joanne Emerson**

United States of America

**Danilo Ercolini**

Italy

**Patricia Fabian**

United States of America

**Jennifer Fettweis**

United States of America

**Roberto Flores**

United States of America

**Anthony Fodor**

United States of America

**Larry Forney**

United States of America

**Kristoffer Forslund**

Germany

**James Foster**

United States of America

**Betsy Foxman**

United States of America

**Pilar Francino**

Spain

**Florian Fricke**

Germany

**Valerie Gaboriau-Rothiau**

France

**Pawel Gajer**

United States of America

**Andrew Gennery**

United Kingdom

**Sommer Gentry**

United States of America

**Philippe Gerard**

France

**Andre Gessner**

Germany

**Sean Gibbons**

United States of America

**Jack Gilbert**

United States of America

**Elizabeth M. Glass**

United States of America

**Frank Oliver Glöckner**

Germany

**Greg Gloor**

Canada

**Filipa Godoy**

Puerto Rico

**Gregor Gorkiewicz**

Austria

**Jan Graffelman**

Spain

**Elizabeth Grice**

United States of America

**Ann Griffen**

United States of America

**Steven Hallam**

Canada

**Jarrad Hampton-Marcel**

United States of America

**Siegfried Hapfelmeier**

Switzerland

**Hermie Harmsen**

Netherlands

**Dennis Hartigan-O’Connor**

United States of America

**Sean Hemmingsen**

Canada

**Matthew Henn**

United States of America

**César Hernández-Rodríguez**

Mexico

**Robert Hettich**

United States of America

**Falk Hildebrand**

Germany

**Janet Hill**

Canada

**Christoph Hoegenauer**

Austria

**Dawn Holmes**

United States of America

**Yuichi Hongoh**

Japan

**Marcus Horn**

Germany

**Denina Hospodsky**

United States of America

**William Hsiao**

Canada

**Gary Huffnagle**

United States of America

**Laura Hug**

United States of America

**Bonnie Hurwitz**

United States of America

**John Huss**

United States of America

**Curtis Huttenhower**

United States of America

**Nico Jehmlich**

Germany

**Timothy Johnson**

United States of America

**Catherine Juste**

France

**Janine Kamke**

New Zealand

**Dae-Kyung Kang**

South Korea

**Bart Keijser**

Netherlands

**Scott Kelley**

United States of America

**Paul Kelly**

United Kingdom

**Alexander Khoruts**

United States of America

**Dan Knights**

United States of America

**Kevin Kohl**

United States of America

**Heidi Kong**

United States of America

**Evguenia Kopylova**

Canada

**Emil Kozarov**

United States of America

**Anita Kozyrskyj**

Canada

**David Kristensen**

United States of America

**Tom Kuntz**

United States of America

**Tak-Wah Lam**

Hong Kong

**Emily Landon**

United States of America

**Andrew Lane**

United States of America

**Morgan Langille**

Canada

**Christian Lauber**

United States of America

**Simon Lax**

United States of America

**Patrick Lee**

Hong Kong

**Charles Lee**

New Zealand

**Jonathan Leff**

United States of America

**Huiying Li**

United States of America

**Yong Li**

United States of America

**Matthew Links**

Canada

**Cindy Liu**

United States of America

**Nathan Lo**

Australia

**Gunnar Loh**

Germany

**Nicholas Loman**

United Kingdom

**Catherine Lozupone**

United States of America

**Michael Lynch**

Canada

**Bing Ma**

United States of America

**Jean Mackaim**

Canada

**Juliette Madan**

United States of America

**Volker Mai**

United States of America

**Stephen Malnick**

Israel

**Mark Manary**

United States of America

**Shannon Manning**

United States of America

**Costa Maranas**

United States of America

**Julian Marchesi**

United Kingdom

**Jessica L. Mark Welch**

United States of America

**Robyn Marsh**

Australia

**Erick Matsen**

United States of America

**Daniel Mcdonald**

United States of America

**Paul Mcmurdie**

United States of America

**David Mead**

United States of America

**James Meadow**

United States of America

**Holly Menninger**

United States of America

**Sam Minot**

United States of America

**Alex Mira**

Spain

**Deborah Money**

Canada

**Emmanuel F. Mongodin**

United States of America

**Ryan Moore**

United States of America

**Michael Morowitz**

United States of America

**Atsushi Nakabachi**

Japan

**David Nelson**

United States of America

**Josef Neu**

United States of America

**Josh Neufeld**

Canada

**Terry Fei Fan Ng**

United States of America

**Per Halkjær Nielsen**

Denmark

**Jens Nielsen**

Sweden

**Kerry Oliver**

United States of America

**Laura Parfrey**

Canada

**John Parkinson**

Canada

**Donovan Parks**

Canada

**Joseph Paulson**

United States of America

**Miia Pitkaranta**

Finland

**Stephen Pointing**

New Zealand

**Philippe Poirier**

France

**Eamonn Quigley**

United States of America

**Julie Renwick**

United States of America

**Frederico Rey**

United States of America

**Alejandro Reyes**

United States of America

**Geraint Rogers**

Australia

**Rohan Sachdeva**

United States of America

**Michael Sadowsky**

United States of America

**Mark Sagoff**

United States of America

**Eric Sakowski**

United States of America

**Stephen Salipante**

United States of America

**Steven Salzberg**

United States of America

**Michael Scharf**

United States of America

**Patrick Schloss**

United States of America

**Herbert Schmidt**

Germany

**Pamela Schnupf**

France

**Clarissa Schwab**

Switzerland

**Anna Seekatz**

United States of America

**Leopoldo Segal**

United States of America

**David Sela**

United States of America

**Awad Shehata**

Germany

**Howard Shuman**

United States of America

**Kathleen Sim**

United Kingdom

**Bärbel Stecher**

Germany

**Garret Suen**

United States of America

**Martin Tàubel**

Finland

**Casey Theriot**

United States of America

**Amanda Thompson**

United States of America

**Ian Toma**

United States of America

**Jaak Truu**

Estonia

**Benjamin Tully**

United States of America

**Matthias Ullrich**

Germany

**David Vallenet**

France

**Marius Vital**

United States of America

**Emily Vogtmann**

United States of America

**William Wade**

United Kingdom

**Honorine Ward**

United States of America

**Eric Webb**

United States of America

**Derrick Wood**

United States of America

**Dongying Wu**

United States of America

**Tanya Yatsunenko**

United States of America

**Vincent Young**

United States of America

**Egija Zaura**

Netherlands

**Fangqing Zhao**

China

**Qi Zheng**

United States of America

**Lifeng Zhu**

China

